# Individuals’ Coping Styles and Levels of Depression, Anxiety, and Stress During the COVID-19 Pandemic in Turkey: A Web-Based Cross-Sectional Study

**DOI:** 10.1177/21582440221148628

**Published:** 2023-01-25

**Authors:** Hilal Altundal Duru, Serpil Yılmaz, Zeliha Yaman, Münevver Boğahan, Mualla Yılmaz

**Affiliations:** 1 Mersin University, Turkey

**Keywords:** COVID-19, depression, anxiety, stress, coping style

## Abstract

This cross-sectional study aimed to determine the correlation between the coping styles and depression, anxiety, and stress levels of individuals living in Turkey during the COVID-19 pandemic. The study was conducted using an online questionnaire (Socio-demographic Form; Depression, Anxiety, and Stress Scale-21; Coping Styles Scale) and it included 483 individuals. Descriptive statistics, ANOVA, Independent Samples *t*-test, Kolmogorov-Smirnov, Hosmer-Lemeshow and Scheffe tests, Pearson Correlation, and Binary Logistic Regression analyzes were used to analyze the data. There was a negative correlation between the participants’ self-confident and optimistic coping styles mean scores and their depression, anxiety, and stress mean scores. There was a positive correlation between the participants’ helpless, submissive, and seeking social support coping styles mean scores and their depression, anxiety, and stress mean scores. The regression analysis revealed that using the helpless coping style increased the depression, anxiety, and stress levels of the participants while using the optimistic coping style and visiting a physician during the pandemic decreased them. In addition, seeking social support coping style increased the level of depression while the testing during the pandemic increased stress levels. As a result, it is recommended to strengthen society’s psychological resilience and expand mental health support services for such mental illnesses.

## Introduction

The conditions in which we live and work are the main factors that affect health and well-being. Economic, social, and environmental changes that are not under the control of the individual pave the way for the development of mental and physical health problems. Mental health is highly sensitive to traumatic events and their social and economic consequences. In addition to physical ilnesses, outbreaks cause neuropsychiatric disorders due to central nervous system involvement and mental symptoms arising from the difficulties of living with the epidemic, independent of the infectious agent. In December 2019, the world faced the reality of a pandemic of Coronavirus Disease-19 (COVID-19) in Wuhan, China (C. Wang et al., 2020). On March 11, 2020, the World Health Organization (WHO), declared COVID-19 a pandemic and a public health emergency of international concern, its “highest level of alarm” (World Health Organization [WHO], 2020). The first confirmed case of COVID-19 in Turkey was reported on 11 March 2020. In Turkey, national data regarding COVID-19 are updated daily, and the total number of cases was 14,978,031 and the number of patients who died due to COVID-19 was 98,493 as of 14 April 2022. There were 500,186,525 confirmed COVID-19 cases, including 6,190,349 deaths, reported to WHO as of 14 April 2022 (WHO, 2022).

Pandemic is a crisis process that threatens the lives and existence of individuals and is distressing for everyone. It has many traumatic effects on individuals, both physically and psychologically (Pfefferbaum & North, 2020). Uncertainty about the course of the disease, isolation and quarantine, separation from loved ones, fear of infecting himself or his family, and loss of freedom can cause psychological problems such as social isolation, loss of income, loneliness, inactivity, limited access to basic services, increased access to food, alcohol, and online gambling, decreased family and social support, irritability, insomnia, anger, avoidance of crowded places, depression, fear, anxiety about not getting adequate and efficient health care, sleep problems, and anxiety (Moreno et al., 2020; Pfefferbaum & North, 2020). Failure to fulfill cultural or religious rituals in the deaths experienced in this period, and tragic consequences such as not being able to say goodbye to their relatives cause the grief process to be postponed and not completed (Pfefferbaum & North, 2020). During the period of social isolation, the feeling of confinement, loss of usual routine, and reduced social and physical contact with others leads to distress, frustration, and feelings of isolation from others (Brooks et al., 2020). Both data from previous pandemics and emerging data from this pandemic suggests the inevitability of a rise in psychological morbidity (Gavin et al., 2020).

In addition, an important psychological factor affecting the impact of stressful life events such as COVID-19 on individuals’ mental health is how individuals cope with stress. Coping is generally defined as the cognitive and behavioral efforts that individuals employ in order to manage stress (Folkman & Moskowitz, 2007). According to the transactional theory of stress and coping (Lazarus & Folkman, 1984), coping occurs in response to stressors, or stimuli in one’s environment that are perceived as threatening, challenging, or harmful (Lazarus & Folkman, 1984). Coping has two main functions: regulating disturbing emotions (aimed at regulating emotional distress) and focusing cognition and behavior on solving the problem that causes distress (aimed at altering person-environment relationships; Lazarus, 1990). Coping strategies play a very important role in physical and mental health, especially during adjustment to stressful situations in life (Endler & Parker, 1994).

As the COVID-19 pandemic is currently one of the largest epidemics worldwide, there is an increase in research focusing on individual coping strategies that can mitigate the occurrence of psychopathologies among the general and quarantined population. Research measuring the styles and levels of coping with depression, anxiety, and stress of individuals during the pandemic is limited (Budimir et al., 2021; Gurvich et al., 2021, Okafor et al., 2022; Skapinakis et al., 2020). Current evidence suggests that there is a need for additional research investigating the relationship between depression, anxiety, and stress levels and coping styles of individuals in the general population during the COVID-19 pandemic. A few studies were conducted with students and healthcare professionals in Turkey. However, none of them examined the stress, depression, anxiety levels, and coping styles of the general Turkish population during the COVID-19 pandemic (Besirli et al., 2021; Özçevik Subaşi et al., 2021; Sümen & Adıbelli, 2021; Türk et al., 2021). This study aims to understand and define the psychosocial responses to the pandemic, the current state of coping strategies and their relationship with demographic variables in order to come up with an understanding of effective coping strategies to minimize psychological symptoms.

Studies conducted in China, the first country affected by the spread of the virus, showed that people’s fear of the unknown nature of the virus could lead to mental disorders (Huang & Zhao, 2020; Salari et al., 2020). In their meta-analysis study, Salari et al. (2020) showed that the COVID-19 pandemic affected the mental health of individuals and communities, and the prevalence of stress (29.6%), anxiety (31.9%), and depression (33.7%) in communities was quite high. A review of the relevant literature in Turkey suggests that currently, there are not any systematic studies on the relationship between Turkish populations’ levels of depression, anxiety, and stress and their preferred coping strategies during the COVID-19 pandemic. Again, based on the review of the existing literature on the subject, we consider this research article as one of the first studies to examine the psychological impacts of COVID-19 on individuals in Turkey and their coping styles. In the current pandemic conditions, it is vital to identify individuals from different groups and populations susceptible to mental disorders and to protect and improve the mental health of the general population with appropriate psychological strategies, techniques, and interventions. The findings of this study could assist government agencies and mental health professionals in protecting the psychological well-being of communities in Turkey and other parts of the world against the potential psychological impact of the COVID-19 pandemic. Since this study is exploratory, starts by presenting the current situation. In doing so, this study aims to determine the correlation between the coping styles and depression, anxiety, and stress levels of individuals living in Turkey during the COVID-19 pandemic and to make comparisons between different groups in this respect.

## Methods

### Study Design

This study was conducted with individuals living in Turkey during the COVID-19 pandemic. The study population included individuals aged 18 years and older who were living in Turkey between August 26 and September 30, 2020, who agreed to participate in the study, and who could speak and understand Turkish. Data were collected during the period when the second peak of the first wave was experienced in the COVID-19 pandemic in Turkey. Because the COVID-19 infection is transmitted through close contact, the study was conducted online. The researchers created an online questionnaire link using Google Forms to prevent the risk of transmission. The study sample was selected using the snowball sampling technique. The first announcement of the survey was made by the researchers through social media posts and messages. In addition, the participants whose survey link was sent via message were asked to share the link with their family, friends, colleagues, and people around them. In addition, some participants also shared the survey link from their own social media accounts.

### Data Collection

Data were collected through a Google Forms questionnaire prepared by the researchers in line with the literature and using the “Socio-demographic Form,” which is a questionnaire on the socio-demographic characteristics and COVID-19 related experiences of the participants, the Depression, Anxiety, and Stress Scale-21 (DASS-21; Lovibond & Lovibond, 1995; Sarıçam, 2018) and the Coping Styles Scale (CSS; Folkman & Lazarus, 1980; N. H. Şahin & Durak, 1995). The forms were shared online between August 26 and September 30, 2020, and the responses of the participants were collected. Several strategies were applied on the web-based data collection form to increase the quality of the questionnaire and to ensure that the participants would give reliable responses to the questions. First of all, since each question was labeled as “Required,” the participants could not submit the form without filling it in. Also, participation of individuals under the age of 18 was prevented and explanations were added to encourage the participants to carefully respond to the questionnaire items. Finally, those questionnaire forms filled in less than 1 minute or more than 60 minutes were excluded from the analysis.

In the online survey form created, the participants were informed about the research on the first page and asked whether they would like to participate in order to obtain their informed consent. The survey forms of the participants who marked the option “I don’t want to participate” were terminated. Therefore, the participants answered the questions voluntarily. While creating the online questionnaire, the age question was asked as an open-ended question. In order for the participants to answer this question both with numbers and to be 18 years or older, this question was stated as “accept numbers only and do not accept numbers below 18” in the settings of the online survey.

### Data Collection Tools

The socio-demographic form was prepared based on the relevant literature. The items in the form were about the participants’ socio-demographic characteristics (e.g., age, gender, level of education, occupation, marital status, level of household income, and chronic disease status) and exposure variables such as consulting a physician, having a test for COVID-19, hospitalization, quarantine, contacting a person with COVID-19, and experiencing COVID-19 symptoms during the pandemic (Huang & Zhao, 2020; C. Wang et al., 2020).

Depression, Anxiety and Stress Scale-21 (DASS-21) is a shorter (21-item) version of the DASS-42 (a 42-item self-report instrument), which was modified by Lovibond and Lovibond (1995). The psychometric properties of the Turkish version of the DASS-21 were examined by Sarıçam (2018) in community and clinical samples, and the scale was found to be valid and reliable. The Cronbach’s alpha internal consistency coefficients of the scale were found as .87 for the depression sub-dimension, .85 for the anxiety sub-dimension, and .81 for the stress sub-dimension. It is a 4-point Likert scale and includes seven items on each of the sub-dimension of “depression, anxiety, and stress.” If a respondent scores 5 points or higher on the depression sub-dimension, 4 points or higher on the anxiety sub-dimension, or 8 points and higher on the stress sub-dimension, this indicates that they have the corresponding problem (Sarıçam, 2018; Table 1)

**Table 1.** Point Values of DASS-21 Sub-Dimensions.

Values	Depression	Anxiety	Stress
Normal	0–4	0–3	0–7
Mild	5–6	4–5	8–9
Middle	7–10	6–7	10–12
Further	11–13	8–9	13–16
Too far	14 and above	10 and above	17 and above

Coping Styles Scale (CSS); was originally developed by Folkman and Lazarus (1980). It was adapted for use in Turkish and modified by N. H. Şahin and Durak (1995) into a shorter 4-point Likert-type self-rating scale consisting of 30 items. The five sub-dimension are optimistic, self-confident, helpless, submissive, and seeking social support coping style. On the scale, items 1 and 9 are reverse scored. The sub-dimensions are scored independently from each other, and it is concluded that the higher the score in a sub-dimension, the more likely it is to use the corresponding coping style. Responses to the items reflect the frequency with which each coping style is used and scored between “0 = Does not apply or not used” and “3 = Used a great deal” (N. H. Şahin & Durak, 1995). There are several reasons for using CSS in this study. First, this scale, which was developed to measure coping mechanisms, has a theoretical background. Additionally, CSS considers individual differences in handling extraordinary situations. That is, the relatively more permanent styles observed in individuals’ coping mechanisms, which occur due to personality or other reasons, may be useful in explaining their coping behaviors. Therefore, CSS was considered appropriate to be used in this study, as it includes behaviors that are more permanent and do not change much from situation to situation, and also benefit from the cognitive and behavioral richness in the coping process (Folkman & Lazarus, 1980).

### Ethical Consideration

Before data collection, all the necessary permissions were obtained from the Ministry of Health and the Ethics Committee (26 August 2020/36). Also, the participants gave written informed consent via the web-based form.

### Data Analysis

Data were analyzed using SPSS 22.0 software. An alpha level of .05 was used as the cut-off for significance. Reliability was checked using Cronbach’s alpha coefficient. The normal distribution of the data was confirmed through the Kolmogorov-Smirnov test. The mean and standard deviation were used for the statistical analysis of continuous values, and frequency and percentage values were used for the statistical analysis of categorical values. Data were analyzed using the Student *t*-test for comparisons between two groups and the analysis of variance (ANOVA) test to compare the means of more than two homogeneous groups. The variables with significant differences between means, according to the ANOVA test results, were further analyzed with the Scheffe post hoc test. The relationship between DASS-21 and CSS sub-dimensions was analyzed by PearsonCorrelation analysis. To determine the variables affecting depression, anxiety, and stress levels of participants, variables that were statistically significant and close to significance in the previous analyzes were tested with Binary Logistic Regression analysis in multivariate analysis. Model fit was evaluated with the Hosmer-Lemeshow test. If the *p*-value in the model is greater than .05, the predictive value of the model is considered high (Alpar, 2016).

## Results

The mean age of the participants was 26.91 ± 7.82 (min: 18, max: 63; the 25th, 50th, and 75th percentiles are 22, 24, and 29, respectively), 72.7% of them were women, 74.9% were single, 63.8% were college graduates, 54% had household income equal to expenditure, 80.5% lived with their families, and 88.2% did not have a chronic disease. According to the participants’ responses, during the pandemic, 23.4% of them knew a person diagnosed with COVID-19 among their family members or friends, 54.9% followed social distancing guidelines strictly, 18.4% visited a physician, 11% had a test for COVID-19, 1% were hospitalized, 5.2% were quarantined, and 10.8% came in contact with the virus (direct or indirect contact with a person diagnosed with COVID-19 or infected material; Tables 2 and 3).

**Table 2.** Sociodemographic Variables of the Participants (*n* = 483).

The socio-demographic variables	*n*	%
Age		
18–25	282	58.4
26 and above	201	41.6
Gender		
Woman	351	72.7
Man	132	27.3
Educational level		
High school-primary school	53	11.0
Associate degree	56	11.6
Graduate	308	63.8
Postgraduate	66	13.7
Profession		
Student	193	40.0
Unemployed-retired	65	12.5
Public sector	90	18.6
Private sector	44	9.1
Academician	36	7.5
Clinician	55	11.4
Marital status		
Married	362	74.9
Single	121	25.1
Household income		
Less than income	157	32.5
Equal	261	54.0
More than income	65	13.5
Cohabitation		
Single	69	14.3
Family	389	80.5
Friend	25	5.2

*Note. n* = number; % = percentage

**Table 3.** Clinical Variables of the Participants (*n* = 483).

Clinical variables	*n*	%
Having a chronic disease		
Yes	57	11.8
No	426	88.2
Visiting a physician during the pandemic		
Yes	89	18.4
No	394	81.6
Testing during the pandemic		
Yes	53	11.0
No	430	89.0
Hospitalizing during the pandemic		
Yes	5	1.0
No	478	99.0
Being quarantined during the pandemic		
Yes	25	5.2
No	458	94.8
Having family members or friends diagnosed with COVID-19		
Yes	113	23.4
No	370	76.6
Following social distancing guidelines		
Never	2	0.4
Sometimes	48	9.9
Quite often	265	54.9
Strictly	168	34.8
Contact with the COVID-19		
Yes	52	10.7
No	431	89.2

*Note. n* = number; % = percentage.

For the DASS-21, the sub-dimension mean scores of the participants for depression, anxiety, and stress were 5.46 ± 4.45, 3.69 ± 3.33, and 6.01 ± 4.12, respectively. The 25th, 50th, and 75th percentiles for depression, anxiety, and stress scores are 2 to 5 to 8, 1 to 3 to 6, 3 to 6 to 9, respectively. It was found that 51.3% of the participants experienced depression, 45.3% had anxiety, and 31.9% had stress. In this study, the Cronbach’s alpha values of the sub-dimension of DASS-21 were found as .88 for the depression sub-dimension, .79 for the anxiety sub-dimension, and .83 for the stress sub-dimension. The mean scores of the participants in the sub-dimension of the CSS were found as 12.67 ± 4.87 in the self-confident coping style, 9.52 ± 4.76 for the helpless coping style, 5.44 ± 3.32 for the submissive coping style, 8.07 ± 3.42 for the optimistic coping style, and 6.36 ± 2.40 for the seeking social support coping style. In this study, the Cronbach’s alpha values of the sub-dimension of CSS were calculated as .83 for the “optimistic coping style” sub-dimension, .90 for the “self-confident coping style” sub-dimension, .79 for the “helpless coping style” sub-dimension, .72 for the “submissive coping style,” and .61 for the “seeking social support” sub-dimension (Table 4).

**Table 4.** Distribution of the Mean Scores of DASS-21 and CSS Sub-Dimensions (*n* = 483).

Scales	Sub-dimension	*n* (%)	Number of items	Minimum-maximum score	$\bar{X}$ ± *SD*	Cronbach’s α
DASS-21	Depression	248 (51.3%)	7	0–21	5.46 ± 4.45	.88
	Anxiety	268 (45.3%)	7	0–21	3.69 ± 3.33	.79
	Stress	154 (31.9%)	7	0–21	6.01 ± 4.12	.83
CSS	Self-confident		7	0–21	12.67 ± 4.87	.90
	Helpless		8	0–24	9.52 ± 4.76	.79
	Submissive		6	0–18	5.44 ± 3.32	.72
	Optimistic		5	0–15	8.07 ± 3.42	.83
	Seeking social support		4	0–12	6.36 ± 2.40	.61

*Note. n* = number; $\bar{x}$ = mean; *SD* = standard deviation.

It was determined that there was no statistically significant difference in the DASS-21 sub-dimension mean scores according to the gender, education level, marital status, and cohabitation variables of the participants (*p* > .05). According to the occupational variable of the participants, the DASS-21 depression sub-dimension mean scores were the highest in students, and the difference between the groups was statistically significant. The Scheffe test was used to determine at the difference between the groups in the depression mean scores. The difference between the “Public sector” and “student” groups was significant (*p* = .029). The DASS-21 depression, anxiety, and stress sub-dimension mean scores of the participants whose income was less than expenses were found to be significantly higher than “Income more than expenses” (respectively, *p* = .022, *p* = .04, *p* = .029). The Scheffe test was used to determine from which group the difference originated (Table 5).

**Table 5.** Data on the Socio-Demographic Variables Affecting DASS-21 Sub-Dimensions Mean Scores of the Participants (*n* = 483).

The socio-demographic variables	DASS-21			
	*n*	Depression	Anxiety	Stress
		$\bar{X}$ ± *SD*	$\bar{X}$ ± *SD*	$\bar{X}$ ± *SD*
Gender				
Woman	351	5.44 ± 4.46	3.66 ± 3.34	5.98 ± 4.11
Man	132	5.53 ± 4.44	3.74 ± 3.30	6.07 ± 4.16
*t*		−0.201	−0.231	−0.202
Cohen’s *d*		0.02	0.02	0.02
*df*	481			
*p*		.841	.817	.840
Educational level				
High school-primary school	53	4.98 ± 3.64	3.58 ± 3.00	5.91 ± 4.03
Associate degree	56	5.13 ± 4.13	3.96 ± 3.68	5.93 ± 3.54
Graduate	308	6.15 ± 5.27	3.59 ± 3.33	5.98 ± 4.16
Postgraduate	66	6.17 ± 4.95	3.95 ± 3.28	6.26 ± 4.52
*F*		0.650	0.612	0.102
*df*	482			
*p*		.460	.777	.959
Profession				
Student	193	6.01 ± 4.82^a^	3.90 ± 3.44	6.54 ± 4.40
Unemployed	65	5.34 ± 4.07	3.49 ± 3.58	6.18 ± 3.98
Public sector	90	4.22 ± 3.50	3.32 ± 2.75	5.29 ± 3.34
Private sector	44	5.91 ± 4.40	4.25 ± 3.62	6.02 ± 4.59
Academician	36	4.58 ± 3.88	2.67 ± 3.36	4.94 ± 4.31
Clinician	55	5.96 ± 4.95	3.96 ± 3.15	5.78 ± 3.72
*F*		2.100	1.209	1.451
Cohen’s *d*		0.440		
df	482			
*p*		.029*	.212	.125
Marital status				
Married	121	5.65 ± 4.30	3.79 ± 3.31	6.40 ± 4.22
Single	362	5.40 ± 4.50	3.65 ± 3.34	5.87 ± 4.08
*t*		−0.539	−0.412	−1.229
Cohen’s *d*		0.05	0.04	0.12
*df*	482			
*p*		.590	.681	.220
Household income				
Less than income	157	6.01 ± 4.65^b^	3.92 ± 3.54^b^	6.48 ± 4.20^b^
Equal	261	5.45 ± 4.40	3.87 ± 3.30	6.01 ± 4.20
More than income	65	4.20 ± 3.95	2.40 ± 2.57	4.86 ± 3.37
*F*		3.825	5.712	3.565
Cohen’s *d*		0.103	0.062	0.084
*df*	482			
*p*		.022*	.004*	.029*
Cohabitation				
Single	69	6.07 ± 4.93	3.81 ± 3.168	6.13 ± 4.01
Family	389	5.31 ± 4.33	3.67 ± 3.381	5.97 ± 4.17
Friend	25	6.16 ± 4.89	3.60 ± 3.082	6.24 ± 3.77
*F*		1.179	0.063	0.087
*df*	482			
*p*		.309	.939	.917

*Note. n* = number; $\bar{x}$ = mean; *SD* = standard deviation; *df* = degrees of freedom; *t* = independent sample *T* test value; *F* = one way ANOVA test value; Cohen’s *d* = effect size.

a Statistically significant difference from the “public sector.”

b Statistically significant difference from the “more than income” group.

* *p* < .05.

The DASS-21 depression sub-dimension mean scores of the participants with chronic disease were found to be significantly higher than those without the chronic disease (*p* = .009). The DASS-21 depression, anxiety, and stress sub-dimension mean scores of the participants who visited a physician during the pandemic were found to be significantly higher than those who din not visit a physician during the pandemic (*p* = .001, *p* < .001, *p* < .001). There was no statistically significant difference between the DASS-21 sub-dimension mean scores and having the testing during the pandemic (*p* > .05). The DASS-21 depression and anxiety sub-dimension mean scores of those hospitalized during the pandemic were significantly higher than those who were not hospitalized (respectively, *p* = .029; *p* < .001). The DASS-21 stress sub-dimension mean scores of the participants who stated that they were in quarantine during the pandemic process were found to be significantly higher than those who were not in quarantine (*p* = .020). The DASS-21 anxiety sub-dimension mean scores of the participants who stated that there were individuals diagnosed with COVID-19 in their family members or friends during the pandemic process were found to be significantly higher than those who did not have anyone diagnosed with COVID-19 among their family members or friends (*p* = .040). The DASS-21 stress mean scores of the participants who stated that they applied social distance measures “quite often” were found to be the highest (*p* = .009). The Scheffe test was used to determine from which group the difference originated. The difference between “Quite often” and “Strictly” groups were significant. The DASS-21 anxiety sub-dimension mean scores of the participants who stated that they had contact with an individual diagnosed with COVID-19 were found to be significantly higher than participants who stated no contact (*p* = .008; Table 6).

**Table 6.** Data on the Clinical Variables Affecting DASS-21 Sub-Dimensions Mean Scores of the Participants (*n* = 483).

Clinical variables	DASS-21			
	*n*	Depression	Anxiety	Stress
		$\bar{X}$ ± *SD*	$\bar{X}$ ± *SD*	$\bar{X}$ ± *SD*
Having a chronic disease				
Yes	57	5.95 ± 4.51	5.07 ± 4.26	6.58 ± 4.44
No	426	5.40 ± 4.44	3.50 ± 3.14	5.93 ± 4.08
*t*		−0.872	−2.685	−1.116
Cohen’s *d*		0.12	0.41	0.15
*df*	481			
* p*		.383	.009**	.265
Visiting a physician during the pandemic				
Yes	89	6.89 ± 4.61	5.33 ± 3.79	7.53 ± 4.52
No	314	5.14 ± 4.36	3.31 ± 3.10	5.66 ± 3.95
*t*		−3.374	−4.656	−3.910
Cohen’s *d*		0.39	0.58	0.44
*df*	481			
* p*		.001**	<.001***	<.001***
Testing during the pandemic				
Yes	53	5.55 ± 5.04	4.13 ± 3.91	5.30 ± 4.09
No	430	5.45 ± 4.38	3.63 ± 2.25	6.09 ± 4.12
*t*		−0.144	−1.035	1.318
Cohen’s *d*		0.02	0.15	0.19
*df*	481			
* p*		.885	.374	.188
Hospitalizing during the pandemic				
Yes	5	9.80 ± 5.63	9.00 ± 5.65	9.60 ± 6.34
No	478	5.42 ± 4.42	3.63 ± 3.26	5.97 ± 4.08
*t*		−2.196	−2.119	−1.964
Cohen’s *d*		0.86	1.16	0.68
*df*	481			
* p*		.029*	<.001***	.051
Being quarantined during the pandemic				
Yes	25	6.96 ± 5.54	5.48 ± 4.88	7.88 ± 5.17
No	458	5.38 ± 4.38	3.59 ± 3.20	5.90 ± 4.04
*t*		−1.398	−1.916	−2.343
Cohen’s *d*		0.31	0.45	0.42
*df*	481			
* p*		.174	.067	.020*
Having family members or friends diagnosed with COVID-19				
Yes	113	5.95 ± 4.47	4.25 ± 3.32	6.50 ± 3.94
No	370	5.32 ± 4.44	3.51 ± 3.32	5.86 ± 4.17
*t*		−1.318	−2.058	−1.442
Cohen’s *d*		0.14	0.22	0.15
*df*	481			
* p*		.188	.040*	.150
Following social distancing guidelines				
Never	2	4.5 ± 4.95	2.0 ± 2.82	5.0 ± 2.82
Sometimes	48	5.83 ± 3.94	3.75 ± 2.95	6.35 ± 3.57
Quite often	265	5.88 ± 4.18	4.00 ± 3.07	6.50 ± 3.89^a^
Strictly	168	4.72 ± 4.92	3.19 ± 3.76	5.14 ± 4.50
*F*		2.266	4.023	1.096
Cohen’s *d*				0.599
*df*	481			
* p*		.061	.085	.009**
Contact with the COVID-19				
Yes	17	7.12 ± 3.49	5.35 ± 3.79	6.65 ± 3.74
No	466	5.37 ± 4.40	3.52 ± 3.17	5.97 ± 4.11
*t*		−1.315	−2.518	−0.486
Cohen’s *d*		0.44	0.52	0.17
*df*	481			
* p*		.080	.008**	.351

*Note. n* = number; $\bar{x}$ = mean; *SD* = standard deviation; *df* = degrees of freedom; *t* = independent sample *T* test value; *F* = one way ANOVA test value; Cohen’s *d* = effect size.

a Statistically significant difference from the “strictly” group.

* *p* < .05. **p < .01. ***p < .001.

There was no statistically significant difference in CSS sub-dimension mean scores according to the gender, education level, occupation, marital status, and cohabitation variables of the participants (*p* > .05). The CSS self-confident and optimistic sub-dimension mean scores of the participants whose income was less than their expenses were significantly lower (respectively, *p* = .013; *p* = .010). The Scheffe test was used to determine from which group the difference originated. The difference between the “Income less than expenses” and “Equal” groups was significant (Table 7).

**Table 7.** Data on the Socio-Demographic Variables Affecting the CSS Sub-Dimensions Mean Scores of the Participants (*n* = 483).

The socio-demographic variables	CSS					
	*n*	Self-confident	Helpless	Submissive	Optimistic	Seeking social support
		$\bar{X}$ ± SD	$\bar{X}$ ± SD	$\bar{X}$ ± SD	$\bar{X}$ ± SD	$\bar{X}$ ± SD
Gender						
Woman	351	12.67 ± 4.80	9.41 ± 4.76	5.32 ± 3.13	8.06 ± 3.40	6.40 ± 2.40
Man	132	12.65 ± 5.07	9.882 ± 4.78	5.76 ± 3.75	8.12 ± 3.49	6.26 ± 2.42
*t*		0.042	−0.838	−1.278	−0.183	0.575
*df*	481					
*p*		.967	.403	.240	.855	.566
Educational level						
High school-primary school	53	12.58 ± 4.64	9.43 ± 3.95	5.43 ± 3.08	8.15 ± 3.23	6.53 ± 2.19
Associate degree	56	13.75 ± 4.33	10.27 ± 4.86	5.59 ± 3.60	8.68 ± 2.94	6.75 ± 2.43
Graduate	308	12.37 ± 5.05	9.44 ± 4.95	5.45 ± 3.30	7.93 ± 3.57	6.21 ± 2.46
Postgraduate	66	13.21 ± 4.50	9.32 ± 4.43	5.30 ± 3.40	8.18 ± 3.24	6.59 ± 2.25
*F*		1.210	0.516	0.415	0.727	0.879
*df*		482				
*p*		.189	.663	.973	.497	.319
Profession						
Student	193	12.53 ± 4.58	10.05 ± 4.53	5.60 ± 3.17	7.99 ± 3.18	6.38 ± 2.17
Unemployed	65	11.43 ± 5.06	9.38 ± 4.67	5.28 ± 3.39	7.31 ± 3.48	5.94 ± 2.65
Public sector	90	13.01 ± 4.82	8.84 ± 4.65	5.49 ± 3.29	8.71 ± 3.50	6.26 ± 2.36
Private sector	44	12.93 ± 6.01	10.41 ± 5.91	6.18 ± 4.39	7.86 ± 4.18	6.73 ± 3.10
Academician	36	14.03 ± 4.13	8.22 ± 5.23	5.03 ± 3.10	8.61 ± 3.18	6.78 ± 2.47
Clinician	55	12.93 ± 5.01	9.09 ± 4.34	4.71 ± 2.85	8.05 ± 3.47	6.40 ± 2.28
*F*		1.323	1.665	1.258	1.321	0.798
*df*	482					
*p*		.163	.114	.305	.184	.508
Marital status						
Married	121	12.57 ± 5.22	9.67 ± 5.84	5.65 ± 3.37	8.22 ± 3.69	6.60 ± 2.57
Single	362	12.70 ± 4.75	9.47 ± 4.74	5.37 ± 3.30	8.02 ± 3.33	6.28 ± 2.34
*t*		0.251	−0.393	−0.803	−0.551	−1.241
*df*	481					
*p*		.802	.694	.423	.582	.215
Household income						
Less than income	157	11.73 ± 5.29^a^	9.65 ± 4.85	5.27 ± 3.21	7.43 ± 3.60^b^	6.06 ± 2.53
Equal	261	13.06 ± 4.62	9.72 ± 4.80	5.65 ± 3.51	8.29 ± 3.33	6.52 ± 2.32
More than income	65	13.34 ± 4.53	8.40 ± 4.31	5.02 ± 2.69	8.77 ± 3.42	6.36 ± 2.40
*F*		4.419	2.101	1.258	4.660	1.796
Cohen’s *d*		0.236			0.191	
*df*	482					
*p*		.013*	.123	.285	.010*	.167
Cohabitation						
Single	69	13.00 ± 4.47	10.28 ± 4.9	5.20 ± 3.29	7.83 ± 3.28	6.75 ± 2.23
Family	389	12.58 ± 4.97	9.41 ± 4.79	5.47 ± 3.34	8.08 ± 3.46	6.28 ± 2.45
Friend	25	13.04 ± 4.36	9.20 ± 3.87	5.76 ± 3.12	8.60 ± 3.24	6.48 ± 1.93
*F*		0.290	1.028	0.302	0.476	1.156
*df*	482					
* p*		.748	.358	.739	.622	.316

*Note. n* = number; $\bar{x}$ = mean; *SD* = standard deviation; *df* = degrees of freedom; *t* = independent sample *T* test value; *F* = one way ANOVA test value; Cohen’s *d* = effect size.

a Statistically significant difference from the “equal.”

b Statistically significant difference from the “more than income” group.

* *p* < .05.

It was determined that there was no statistically significant difference in the mean CSS sub-dimension scores of the participants according to the chronic disease, visiting a physician during the pandemic, hospitalizing during the pandemic, and being quarantined during the pandemic and contact with the COVID-19 variables (*p* > .05). The CSS seeking social support sub-dimension mean scores of the participants who stated that they had testing during the pandemic were significantly lower than the participants who did not testing (*p* = .029). The CSS helpless sub-dimension mean scores of the participants who stated that there was an individual diagnosed with COVID-19 in their family or among their friends during the pandemic process were significantly higher than the participants who did not have anyone diagnosed with the disease among their family or among their friends (*p* = .016). The CSS self-confident and optimistic sub-dimension mean scores of the participants who stated the status of applying the social distance measures as “never” were the lowest (respectively, *p* = .035; *p* = .022). The Scheffe test was used to determine from which group the difference originated. The difference between the “Quite often” and “Strictly” groups was significant (Table 8).

**Table 8.** Data on the Clinical Variables Affecting the CSS Sub-Dimensions Mean Scores of the Participants (*n* = 483).

Socio-demographic variables	CSS					
	*n*	Self-confident	Helpless	Submissive	Optimistic	Seeking social support
		$\bar{X}$ ± *SD*	$\bar{X}$ ± *SD*	$\bar{X}$ ± *SD*	$\bar{X}$ ± *SD*	$\bar{X}$ ± *SD*
Visiting a physician during the pandemic						
Yes	89	13.04 ± 4.70	10.28 ± 4.44	5.53 ± 3.21	8.20 ± 3.38	6.67 ± 2.26
No	394	12.58 ± 4.91	9.35 ± 4.82	5.42 ± 3.34	8.05 ± 3.43	6.29 ± 2.43
*t*		−0.810	−1.666	−0.267	−0.389	−1.364
*df*	481					
*p*		.418	.096	.789	.697	.173
Testing during the pandemic						
Yes	53	11.89 ± 5.27	8.92 ± 5.27	5.47 ± 3.36	7.66 ± 3.82	5.68 ± 2.40
No	430	12.76 ± 4.82	9.60 ± 4.70	5.44 ± 3.31	8.13 ± 3.37	6.44 ± 2.39
*t*		1.235	0.967	−0.066	0.932	2.193
Cohen’s *d*						0.262
*df*	481					
*p*		.217	.334	.947	.352	.029*
Hospitalizing during the pandemic						
Yes	5	14.40 ± 5.17	13.20 ± 6.87	8.60 ± 6.14	10.00 ± 3.74	8.00 ± 3.00
No	478	12.65 ± 4.87	9.48 ± 4.73	5.41 ± 3.27	8.05 ± 3.42	6.34 ± 2.39
*t*		−0.799	−1.738	−2.145	−1.264	−1.534
*df*	481					
*p*		.425	.083	.311	.207	.126
Being quarantined during the pandemic						
Yes	25	13.08 ± 4.74	10.92 ± 4.72	6.28 ± 3.90	8.00 ± 3.32	6.36 ± 2.11
No	458	12.64 ± 4.88	9.45 ± 4.76	5.40 ± 3.28	8.08 ± 3.43	6.36 ± 2.42
*t*		−0.435	−1.508	−1.295	0.112	0.001
*df*	481					
*p*		.664	.132	.196	.911	1.000
Having family members or friends diagnosed with COVID-19						
Yes	113	13.31 ± 4.71	10.47 ± 4.69	5.88 ± 3.62	8.35 ± 3.25	6.72 ± 2.51
No	370	12.47 ± 4.91	9.23 ± 4.75	5.31 ± 3.21	7.99 ± 3.47	6.25 ± 2.36
*t*		−1.605	−2.426	−1.587	−0.959	−1.804
Cohen’s *d*			0.317			
*df*	481					
*p*		.109	.016*	.113	.338	.072
Following social distancing guidelines						
Never	2	9.00 ± 5.65	4.50 ± 2.12	2.0 ± 2.82	7.50 ± 3.53	4.00 ± 1.41
Sometimes	48	11.56 ± 4.22	10.42 ± 4.12	5.54 ± 2.37	7.58 ± 3.00	6.35 ± 2.05
Quite often	265	12.40 ± 4.86^a^	9.62 ± 4.56	5.43 ± 3.21	7.75 ± 3.41^b^	6.38 ± 2.37
Strictly	168	13.44 ± 4.97	9.17 ± 5.21	5.48 ± 3.70	8.73 ± 3.48	6.36 ± 2.55
*F*		1.829	1.944	2.019	0.907	1.515
Cohen’s *d*		0.354			0.474	
*df*	482					
*p*		.035*	.178	.529	.022*	.585
Contact with the COVID-19						
Yes	17	14.29 ± 4.45	10.76 ± 4.95	7.12 ± 3.83	9.29 ± 3.19	7.41 ± 2.45
No	466	12.67 ± 4.81	9.48 ± 4.80	5.36 ± 3.28	8.06 ± 3.38	6.35 ± 2.38
*t*		0.050	−0.581	−1.548	−0.305	−0.382
*df*	481					
*p*		.141	.122	.110	.437	.210

*Note. n* = number; $\bar{x}$ = mean; *SD* = standard deviation; *df* = degrees of freedom; *t* = independent sample *T* test value; *F* = one way ANOVA test value; Cohen’s *d* = effect size.

a Statistically significant difference from the “strictly” group.

b Statistically significant difference from the “strictly” group.

* *p* < .05.

There was a weak, negative significant correlation between the participants’ age and their depression, anxiety, and stress sub-dimension mean scores (respectively, *r* = −.204, −.133, and −.181; *p* < .001). However, there was no significant relationship between the age of the participants and the CSS sub-dimensions (*p* > .05). There was a weak negative significant correlation between the participants’ self-confident coping style sub-dimension mean scores and their depression and stress sub-dimension mean scores (respectively, *r* = −.173 and −.138; *p* < .001). There was a strong negative significant correlation between their self-confident coping style sub-dimension mean scores and their anxiety sub-dimension mean scores (*r* = −.900; *p* < .05). There was a moderate, positive significant correlation between the participants’ helpless coping style sub-dimension mean scores and their depression, anxiety, and stress sub-dimension mean scores (respectively, *r* = .507, *r* = .402, *r* = .466; *p* < .001). There was a weak positive significant correlation between the participants’ submissive coping style mean scores and their depression, anxiety, and stress sub-dimension mean scores (respectively, *r* = .296, *r* = .254, *r* = .250; *p* < .001). There was a weak negative significant correlationbetween the participants’ optimistic coping style sub-dimension mean scores and their mean scores in the depression, anxiety, and stress sub-dimension (respectively, *r* = −.212, −.123, and −.209; *p* < .001). There was also a weak positive significant correlation between the participants’ seeking social support coping style sub-dimension mean scores and their mean scores in the depression, anxiety, and stress sub-dimension (respectively, *r* = .167, *r* = .146, *r* = .150; *p* < .01; Table 9).

The regression model that emerged in the Binary Logistic Regression analysis between participants’ depression levels and sociodemographic variables-CSS sub-dimensions was significant (χ^2^ = 151.548, *p* < .05). Using these variables, the created model estimated the participants’ depression levels by 74.1%. While “helpless” coping style increased the depression levels of participants 1.2 times, “seeking social support” coping style increased that level 1.1 times. While “optimistic” coping style decreased the participants’ depression levels 0.7 times, visiting a physician during the pandemic decreased the same level 0.4 times. On the other hand, the difference between participants’ anxiety levels and sociodemographic variables-CSS sub-dimensions were significant (χ^2^ = 81.268, *p* < .05). Using these variables, the created model estimated the participants’ anxiety levels by 67.5%. While “helpless” coping style increased the participants’ anxiety levels 1.1 times, “optimistic” coping style decreased their anxiety levels 0.8 times and visiting a physician during the pandemic decreased the same level 0.4 times. Finally, the difference between the participants’ stress levels and sociodemographic variables-CSS sub-dimensions were significant (χ^2^ = 122.571, *p* < .05). Using these variables, the created model estimated the participants’ anxiety levels by 72.7%. “Helpless” coping style increased the participants’ stress levels 1.2 times and having testing during the pandemic increases the same level 3.1 times. Finally, “optimistic” coping style decreases the participants’ stress levels 0.7 times and visiting a physician during the pandemic decreased that level 0.2 times For depression, Nagelkerke *R*^2^ = .359; *p* is <.05; the correct estimation rate of the model is 74.1%. In Hosmer–Lemeshow test the *p* value of the model is .349 and the predictive value of the model is high. Since the p value of the model is greater than .05, it has sufficient fit. For anxiety, Nagelkerke *R*^2^ = .207; *p* is <.05; the correct estimation rate of the model is 67.5%. In Hosmer–Lemeshow test the *p* value of the model is .051, and the predictive value of the model is high. Since the *p* value of the model is greater than .05, it has sufficient fit. For stress, Nagelkerke *R*^2^ = .314; *p* is <.05; the correct estimation rate of the model is 72.7%. In Hosmer–Lemeshow test the *p* value of the model is .225 and the model has a high predictive value. Since the *p* value of the model is greater than .05, it has sufficient fit (Table 10).

**Table 9.** The Correlation Analysis Between Age, DASS-21, and CSS Sub-Dimensions Mean Scores of the Participants (*n* = 483).

			DASS-21			CSS				
Scales		Age	Depression	Anxiety	Stress	Self-confident	Helpless	Submissive	Optimistic	Seeking social support
DASS-21	Depression	−0.204**	1	0.684**	0.780**	−0.173**	0.507**	0.296**	−0.212**	0.167**
	Anxiety	−0.133		1	0.741**	−0.900*	0.402**	0.254**	−0.123**	0.146**
	Stress	−0.18			1	−0.138**	0.466**	0.250**	−0.209**	0.150**
CSS	Self-confident	0.043				1	0.100*	0.204*	0.852**	0.537**
	Helpless	−0.062					1	0.653**	0.125**	0.509**
	Submissive	0.023						1	0.283**	0.401**
	Optimistic	0.079							1	0.531**
	Seeking social support	0.063								1

* *p* < .05. **p < .001.

**Table 10.** Logistic Regression of Variables Affecting Participants’ Depression, Anxiety, and Stress Levels.

Variables (reference)	Depression				Anxiety				Stress			
	95% confidence interval for Exp^a^ (β)^2^				95% confidence interval for Exp^a^ (β)^2^				95% confidence interval for Exp^a^ (β)^2^			
	β	OR	Min-max	*p*-Value	β	OR	Min-max	*p*-Value	β	OR	Min-max	*p*-Value
Gender (woman)	.268	1.308	0.814−2.101	.267	.028	1.028	0.661−1.600	.902	.157	1.170	0.708− 1.933	.541
Self-confident	−.020	0.980	0.908−1.058	.600	.007	1.007	0.939−1.080	.843	.016	1.016	0.941−1.098	.681
Helpless	.229	1.257	1.168−1.353	.000*	.128	1.137	1.067−1.211	.000*	.212	1.236	1.147−1.331	.000*
Submissive	−.006	0.994	0.907−1.088	.889	.057	1.058	0.974−1.150	.181	−.038	0.962	0.878−1.055	.412
Optimistic	−.253	0.776	0.691−0.872	.000*	−.148	0.863	0.779−0.956	.005*	−.243	0.784	0.697−0.882	.000*
Seeking social support	.139	1.149	1.007−1.312	.039*	.015	1.015	0.901−1.144	.806	.065	1.067	0.934−1.220	.340
Visiting a physician during the pandemic (yes)	−.865	0.421	0.225−0.789	.007*	−.894	0.409	0.232−0.723	.002*	−1.359	0.257	0.140−0.473	.000*
Testing during the pandemic (yes)	.799	2.223	0.973−5.077	.058	.429	1.536	0.713−3.308	.273	1.154	3.170	1.260−7.976	.014*
Hospitalizing during the pandemic (yes)	−1.329	0.265	0.021−3.328	.303	−1.041	0.353	0.031−3.962	.399	−.621	0.537	0.068−4.221	.555
Being quarantined during the pandemic (yes)	.619	1.856	0.674−5.112	.231	.017	1.017	0.386−2.677	.973	−.341	0.711	0.263−1.926	.502
Contact with the COVID-19 (yes)	−.396	0.673	0.313−1.449	.312	−.594	0.552	0.269−1.132	.105	−.429	0.651	0.297−1.427	.284

*Note*. Bold values indicate statistically significant *p* < .05. Nagelkerke *R*^2^: Displays the percentage of the explained variance of the dependent variable. β = regression coefficient; OR = odds ratio; Min = minimum; Max = maximum. Bold values with “*” indicate statistically significant *p* < .05.

a 95% Confidence interval for Exp(β).

## Discussion

COVID-19 has a serious impact on the mental health of communities. This study explored the relationship between the stress-coping styles and depression, anxiety, and stress levels of individuals living in Turkey during the COVID-19 pandemic. . In this study, 51.3% of the participants had depression, 45.3% had anxiety, and 31.9% had stress. Brooks et al. (2020) examined mental disorders during the COVID-19 pandemic and found that affected individuals exhibit various signs of mental trauma such as emotional distress, depression, stress, mood swings, irritability, and insomnia. Similarly, recent studies showed that COVID-19 has effects on mental health such as anxiety, depression, and stress symptoms (Tareke et al., 2022; Y. Wang et al., 2021). Similar observations were reported in a few studies although the percentages of depression, anxiety, and stress in populations differed in these studies. For example, while depression and stress were not observed in Iranian society during the pandemic, the rate of anxiety was 50.9% (Moghanibashi-Mansourieh, 2020). In terms of depression, anxiety, and stress levels by country, these levels were reported, respectively, as 30.3%, 36.4%, and 32.1% in China (Zhou et al., 2020); 30.3%, 36.4%, and 32.1% among residents of Tepi town-Ethiopia (Tareke et al., 2022); 38.9%, 43%, and 35.7% in India (Kazmi et al., 2020); 40%, 60%, and 35% in Jordanian healthcare workers (Alnazly et al., 2021), and 32.8%, 18.7%, and 27.2% in Italy (Mazza et al., 2020). These differences could be related to the unique characteristics of each research sample and location. They might have been caused by potential regional dissimilarities, cultural differences, existing social support systems, social policies followed during the pandemic process, and the methodologies of those studies.

It is vital to have effective coping strategies for stressful situations as these can prevent experiences that lead to stress-related psychiatric disorders. While individual vulnerability to stress and certain situations exist, the use of coping strategies is expected to help (Centers for Disease Control and Prevention [CDC], 2020; WHO, 2020). In this study, the most common coping mechanism, used by the participants was the self-confident coping style, followed by helpless, optimistic, seeking social support, and submissive coping styles. In a study conducted in China, 70.2% of the participants reported that they tried to cope positively with the pandemic by participating in activities, talking to others about concerns, and thinking positively, while 29.8% used passive coping styles such as escaping, smoking, and being dependent on others (Fu et al., 2020). Individuals use ineffective coping strategies such as alcohol and cigarette consumption, as well as positive thinking, seeking social support, and effective stress coping strategies in this process (Budimir et al., 2021). These results suggest that positive coping is a factor that increases resilience. Promoting positive and adaptive coping strategies by educating society about psychological coping mechanisms could further contribute to the development of resilience during the pandemic. Therefore, it is crucial to screen individuals with high levels of stress at an early stage to improve physical and psychological health with timely appropriate psychological interventions. In this study, students’ depression mean scores were found to be significantly higher. Students are more at risk in terms of depression during the COVID-19 process. In the study conducted by Solomou and Constantinidou (2020) to determine the psychosocial effects of the COVID-19 pandemic on the general population, women, young individuals, students, unemployed, those with a psychiatric history and those who report more negative effects on their quality of life are at higher risk for anxiety and depression symptoms. A study evaluating the prevalence of depression, anxiety, and stress symptomatology among university students suggest that sudden changes in teaching methods and workload cause depression and anxiety disorders among students (Fawaz & Samaha, 2021). Similarly, university students’ mental health is adversely affected as they are in the vulnerable group, and they already showed depressive symptoms during the pandemic (Faisal et al., 2022; Lee et al., 2021; D. Wang et al., 2022).

In this study, the DASS-21 depression, anxiety, and stress sub-dimension mean scores of the participants whose income is less than their expenses were found to be significantly higher. Income is also among the sociodemographic characteristics that affect mental health during the COVID-19 process (Fukase et al., 2021; Hossain et al., 2020; Nagasu et al., 2021). In the study by Cao et al. (2020) with university students, regular income is shown among the protective factors against anxiety. The perceived stress is higher and depressive symptoms are seen in low-income individuals during the COVID process (Duan et al., 2020). Also, the change in income during the pandemic process is an important risk factor for depression and hopelessness and causes the mental status of individuals to be negatively affected (Akova et al., 2022).

In this study, the depression, anxiety, and stress mean scores of the participants who visited a physician during the pandemic process were found to be significantly higher. Also, the depression mean scores of the participants with chronic diseases were found to be significantly higher. Evidence suggests an association between medical history and the increased anxiety and depression caused by the spread of COVID-19 (Mazza et al., 2020). Medical history and chronic illnesses are associated with increased levels of psychiatric distress (Akova et al., 2022; Holmes et al., 2020; C. Wang et al., 2020). Research shows that individuals with a history of chronic diseases (physical or mental) before the pandemic experience more depression and psychological problems (Akova et al., 2022; Ozamiz-Etxebarria et al., 2020; Stanton et al., 2020; Wu et al., 2020). The depression and anxiety mean scores of those hospitalized during the pandemic period were found to be significantly higher. Zandifar et al. (2020) reported that the prevalence of psychiatric disorders was high among hospitalized COVID-19 patients, and 97.2% of their patients were depressed, 100% were anxious, and 97.1% had stress. The stress sub-dimension mean scores of the participants who stated that they followed the social distance measures “quite often” and were in quarantine during the pandemic were found to be significantly higher. Within the framework of the measures taken, individuals who came into contact with the infection in many countries isolated themselves at home or in a special quarantine facility. Quarantine causes negative psychological effects such as post-traumatic stress symptoms, anxiety, confusion, and anger. In addition, stressors included long quarantine periods, fears of infection, frustration, boredom, insufficient material, insufficient information, financial loss, and stigma (Brooks et al., 2020). It is also associated with increased stress, anxiety, and depression as days spent in quarantine increase (Shah et al., 2021). In this study, the anxiety mean scores of the participants who stated that there were individuals diagnosed with COVID-19 in their family members or friends and had contact with an individual diagnosed with COVID-19 during the pandemic process were found to be significantly higher. Anxiety scores of those having family members, friends, or acquaintances infected with the disease are significantly higher (Cao et al., 2020; Salman et al., 2020). The risk factors for mental health problems include living in rural areas, being female, and being at risk of exposure to COVID-19 (M. K. Şahin et al., 2020; Zhang et al., 2020). One of the important factors affecting mental health is being at risk of exposure to COVID-19 (Tareke et al., 2022). Anxiety levels are significantly higher in people who have at least one family member, relative, or friend diagnosed with COVID-19 (Cao et al., 2020; Moghanibashi-Mansourieh, 2020; Tareke et al., 2022; C. Wang et al., 2020).

In this study, the self-confident and optimistic sub-dimension mean scores of the participants whose income is less than their expenses were found to be significantly lower. The self-confident and optimistic coping styles of low-income participants were negatively affected during the COVID-19 process. It is vital to have effective coping strategies for stressful situations as these can prevent experiences that lead to stress-related psychiatric disorders. While individual vulnerability to stress and certain situations exist, the use of coping strategies is expected to help (CDC, 2020; WHO, 2020). In a study conducted in China, 70.2% of the participants reported that they tried to cope positively with the pandemic by participating in activities, talking to others about concerns, and thinking positively, while 29.8% used passive coping styles such as escaping, smoking, and being dependent on others (Fu et al., 2020). In the study of Zhang et al. (2020), the irritability level of youths with a lower monthly household income was higher. Irritability of the youth had significantly negative correlations with a positive response, and it had a significantly positive correlation with a negative response. In Park et al.’s (2020) study, the participants experienced high levels of financial anxiety and the most frequently used coping methods were a distraction, active coping, and emotional social support seeking. Also, people experience economic problems during the pandemic process and have difficulties in coping with this situation (Nath et al., 2022).

In this study, the mean scores of seeking social support of the participants who stated that they had testing during the pandemic process were significantly lower. Having a COVID-19 test can negatively affect individuals’ coping styles in seeking social support. The helplessness mean scores of the participants who stated that there was an individual diagnosed with COVID-19 in their family or among their friends during the pandemic process were found to be significantly higher. The presence of a person diagnosed with COVID-19 in her family or among friends during the pandemic process increases helpless coping styles. In a study conducted with health professionals, it was reported that most participants used avoidance coping strategy and participants whose mental health was negatively affected were more likely to adopt seeking social support as a coping strategy (Tahara et al., 2020). In this study, the self-confident and optimistic sub-dimension mean scores of the participants who stated the status of applying social distance measures as “never” were the lowest. The participants who did not obey the social distance rules use less self-confident and optimistic coping strategies. As a result of social distance practices and quarantine in the COVID-19 pandemic, loneliness negatively affected self-confidence and optimistic approach in individuals (Killgore et al., 2020).

These results showed that as the participants’ depression, anxiety, and stress levels increased, their level of using the “helpless,” “submissive,” and “seeking social support” coping styles increased, but their level of using the “optimistic” and “self-confident” coping styles decreased. As a result of the regression analysis, it was revealed that using the helpless coping style increased the depression, anxiety, and stress levels of the participants while using the optimistic coping style and visiting a physician during the pandemic decreased them. In addition, seeking social support coping style increased the level of depression and having the testing during the pandemic increased stress levels. In Kar et al.’s (2021) study, the most common coping mechanisms used by the participants during the pandemic were hoping for the best, remaining busy, and having faith in God. The study also showed that avoiding thinking about the current stressful situation, being unaware of coping strategies, and struggling to cope are significantly associated with anxiety and depression. Yan et al. (2021) found that individuals who adopt mostly positive coping strategies experience less depression and anxiety under stress, while negative coping strategies exacerbate emotional distress. In this respect, it is essential to inform individuals about effective coping strategies against COVID-19 and similar social crises and to take initiatives to support their development of coping skills (Kar et al., 2021; Polizzi et al., 2020). In addition, psychoeducational arrangements by health professionals that will increase the self-confidence, and optimistic perspectives of individuals and reduce depression, anxiety, and stress will be effective during the COVID-19 process. It would be appropriate to include topics such as self-knowledge, emotion management, and coping with stress and anxiety within the scope of psychoeducation. Worries may weigh heavily on individuals as economic difficulties, job losses, and secondary stresses related to grief emerge. The public needs to be informed about available resources and practical methods to deal with these problems that come along with the ongoing stress of COVID-19. In Turkish culture, people traditionally support each other, reflecting the saying, “He who sleeps on a full stomach whilst his neighbor goes hungry is not one of us.” Therefore, individuals in Turkey could have helped each other materially and spiritually during this pandemic. In a sense, people could have tried to overcome this process by healing themselves and each other. Having social support, individuals living in Turkey might have experienced relatively fewer mental problems during the COVID-19 pandemic due to this characteristic of Turkish culture. In this study, a negative significant correlation was found between the participants’ age and their depression, anxiety, and stress sub-dimension mean scores.However,results from some other studies suggest that as the age increases, anxiety, depression, and stress levels increase significantly during the pandemic process (Huang & Zhao, 2020; Moghanibashi-Mansourieh, 2020).

## Conclusions

The coping styles used most by the participants in this study was self-confident coping style, followed by helpless, optimistic, seeking social support, and submissive coping styles. As the age of the participants decreases, their depression, anxiety, and stress levels increase. As the participants” self-confident and optimistic coping styles decrease, their depression, anxiety, and stress levels increase. As the helpless, submissive and social support-seeking coping styles of the participants increase, their depression, anxiety, and stress levels also increase. According to the results of the study, students and the participants with chronic diseases are at risk for depression, those with low income and those who visit a physician during the pandemic are at risk for depression, anxiety, and stress. Those who are in quarantine and who apply social distance measures quite frequently are more at risk in terms of stress, those with family members or friends diagnosed with COVID-19, and those who report having contact with someone diagnosed with COVID-19 are more at risk for anxiety. The results from this study showed that those with less income than their expenses had difficulties in coping with self-confidence and optimism. Also, those who stated that they had been tested during the pandemic period avoided seeking social support. Those who stated that there was an individual diagnosed with COVID-19 in their family or among friends during the pandemic process had a lower mean of helplessness. The participants who never followed the social distance rules showed less self-confident and optimistic attitudes. As a result of the regression analysis, it was revealed that using the helpless coping style increased the depression, anxiety, and stress levels of the participants while using the optimistic coping style and visited a physician during the pandemic decreased them. In addition, seeking social support coping style increased the level of depression and the testing during the pandemic increased stress levels.

During the COVID-19 process, healthcare professionals should consider individuals who are at risk, especially mentally (students, those with chronic disease, those whose income is less than their expenses, those who visited a physician during the pandemic, those who are in quarantine, those who apply social distance measures quite often, those who have members of their family or friends with a diagnosis of COVID-19, and those who state that they have contacted someone with a diagnosis of COVID-19), in their health interventions. Furthermore, it would be appropriate for healthcare professionals to monitor early symptoms of depression, anxiety, and stress in individuals in younger age groups. Psychoeducational arrangements by health professionals that will increase the self-confidence, and optimistic perspectives of individuals and reduce depression, anxiety, and stress will be effective during the COVID-19 process. It would be appropriate to include topics such as self-knowledge, emotion management, and coping with stress and anxiety within the scope of psychoeducation. Studies are needed on the prevalence of mental health problems in Turkish society during the pandemic process, and mental health problems should be addressed with correct planning and structured approaches. As a result, it is recommended to strengthen of the society’s psychological resilience and expand mental health support services for in such mental illnesses.

### Limitations

This study has a few limitations. Firstly, as it is a cross-sectional study, it would be informative to monitor the dynamic change in individuals’ mental health during the COVID-19 pandemic. Secondly, since all the questionnaire forms were collected online by self-reporting, the typical limitations of this process are also present in this study. In addition to this, the biases of the participants, filling in the online questionnaire via self-report and as well as issues specifically with inferring causality from cross-sectional data may have affected the results of the study. Finally, because the majority of individuals participating in the study are young, the sample may be unlikely to be representative of the general population.
